# Supplementary material for: Installation of synergistic binding sites onto porous organic polymers for efficient removal of perfluorooctanoic acid
Source: Nat Commun. 2022 Apr 19;13:2132. doi: 10.1038/s41467-022-29816-1 (PMC9019033; doi:10.1038/s41467-022-29816-1)
Supplement: Supplementary file 1 — Supplementary Information [file 41467_2022_29816_MOESM1_ESM.pdf]

Supplementary Information

Installation of Synergistic Binding Sites onto Porous Organic Polymers for Efficient Removal of Perfluorooctanoic Acid

Xiongli Liu[1], Changjia Zhu[2], Jun Yin[3,4], Jixin Li[5], Zhiyuan Zhang[1], Jinli Li[1], Feng Shui[1], Zifeng You[1], Zhan Shi[5], Baiyan Li[*,1], Xian-He Bu[*,1], Ayman Nafady[6] and Shengqian Ma[*,2]

[1]School of Materials Science and Engineering, National Institute for Advanced Materials, TKL of Metal and Molecule-Based Material Chemistry, Nankai University, Tianjin 300350, P. R. China; [2]Department of Chemistry, University of North Texas 1508 W Mulberry St, Denton, TX 76201 (USA); [3]Physical Sciences and Engineering Division, King Abdullah University of Science and Technology, Advanced Membranes and Porous Materials Center, Thuwal, 23955-6900; [4]Kingdom of Saudi Arabia; KAUST Catalysis Center, Physical Sciences and Engineering Division, King Abdullah University of Science and Technology, Thuwal, 23955-6900, Kingdom of Saudi Arabia; [5]State Key Laboratory of Inorganic Synthesis and Preparative Chemistry, College of Chemistry, Jilin University, Changchun 130012, P. R. China; [6]Department of Chemistry, College of Science, King Saud University, Riyadh 11451, Saudi Arabia

E-mail: libaiyan@nankai.edu.cn; buxh@nankai.edu.cn; Shengqian.Ma@unt.edu

Table of Contents

Section 1. Synthetic Procedures S3

Section 2. Test of chloride content in PAF-1-NDMB S6

Section 3. Nonlinear pseudo second order kinetics model S6

Section 4. Regeneration Experiments S6

Section 5. Supplementary Tables S7

Section 6. Supplementary Figures S9

Section 7. References S28

Section 1. Synthetic Procedures

Synthesis of Tetraphenylmethane. Tetraphenylmethane was synthesized according to the procedure reported by the literature.[1] A 1 L round bottomed flask was charged with chlorotriphenylmethane (97%, 50.0 g, 179.4 mmol) and aniline (99.5%, 44.0 mL, 45.0 g, 46.4 mmol). The reaction mixture was slowly heated to 190 ℃ under vigorous stirring. After 15 min, the reaction mixture was allowed to cool to room temperature. Then, a solution of aqueous HCl (36～38%, 2 M, 200 mL) and methanol (98%, 300 mL) were added to the pulverized solid and the reaction mixture was heated to 80 ℃ for 30 min. After being cooled to room temperature, the resulting solid was filtered off, washed with water, and dried in vacuo (70 ℃, 18 h). In a 1 L round bottomed flask, the dry solid was suspended in N,N-Dimethylformamide (DMF, 99.8%, 500 mL) and cooled to -15 ℃. At this temperature, sulfuric acid (96%, 55 mL) and isoamyl nitrite (95%, 39.8 mL, 34.6 g, 296 mmol) were added slowly and the resulting suspension was stirred for 1 h. Then hypophosphoric acid (50%, 90 mL) was added dropwise. Once the addition was completed, the reaction mixture was heated to 50 ℃ until the evolution of gas had ceased. Then, the solid was filtered off and washed subsequently with DMF, water, and ethanol. This washing procedure was repeated twice. After drying in vacuo (70 ℃, 18 h), tetraphenylmethane was obtained as a brown powder (55.1 g, yield: 96%). ^{1}H NMR (400MHz, $CDCl_3$) δ: 7.17-7.06 (m, 20H). ^{13}C NMR (101 MHz, $CDCl_3$) δ = 146.8, 131.2, 127.5, 125.9. HRMS (m/z): $[M]^+$ calcd. for $C_{25}H_{20}$, 320.4344; found, 320.1559. IR: 3085, 3055, 3029, 3016, 1591, 1491, 1439 cm^{-1}.

Synthesis of Tetrakis(4-bromophenyl)methane. Tetrakis(4-bromophenyl)methane was synthesized according to the procedure reported by the literature.[1] A 1 L three-necked

round-bottomed flask containing bromine (128.0 mL, 398.0 g, 2.5 mol), tetraphenylmethane (40.0 g, 124.8 mmol) was added in small portions under vigorous stirring at room temperature. After the addition was completed, the resulting solution was stirred for 20 min and then cooled to -78 ℃. At this temperature, ethanol (99.5%, 300 mL) was added slowly and the reaction mixture was allowed to warm to room temperature overnight. After this, the precipitate was filtered off and washed subsequently with saturated aqueous sodium hydrogen sulfite solution and water. After drying in vacuo, tetrakis(4-bromophenyl) methane (TBPM) was obtained as a yellow solid (78.85 g, yield: 99.6%). ^{1}H NMR (400MHz, $CDCl_3$) δ: 7.39 (d, J = 8.7 Hz, 8H), 7.01 (d, J = 8.7 Hz, 8H). ^{13}C NMR (101 MHz, $CDCl_3$) δ = 144.5, 132.4, 131.1, 120.8, 63.7. HRMS (m/z): $[M]^+$ calcd. for $C_{25}H_{16}Br_4$, 636.0105; found, 635.7939. IR: 1569, 1479, 1079, 1009, 950, 914, 835, 810, 752, 729, 679 cm^{-1}.

Synthesis of PAF-1. PAF-1 was synthesized according to the procedure reported by the literature.[2] Briefly, Tetrakis(4-bromophenyl)methane (TBPM, 1.02 g, 1.60 mmol) was added to a solution of 2,2’-bipyridyl (99%, 1.13 g, 7.30 mmol), bis(1,5-cyclooctadiene)nickel(0) ($Ni(cod)_2$, 97%, 1.50 g, 5.47 mmol), and 1,5-cyclooctadiene (cod, 99%, 0.90 mL, 7.30 mmol) in the mixture of 120 mL anhydrous dimethylformamide (DMF) and 180 mL anhydrous tetrahydrofuran (THF), and the mixture was stirred overnight at room temperature under nitrogen atmosphere. After the reaction, 6 M HCl (120 mL) was added slowly, and the resulting mixture was stirred for 12 h. The precipitate was collected by filtration, then washed with methanol and water, and dried at 150 ℃ for 24 h under vacuum to produce PAF-1 as an white powder (yield: 80%). Elemental Analysis: C: 92.53%; H: 4.82%. ^{13}C CP-MAS NMR: 146, 140, 131, 125, and 64 ppm. IR: 3030, 1498, 1004, 812, 705 cm^{-1}.

Synthesis of PAF-1-CH_2Cl. PAF-1-CH_2Cl was synthesized according to the procedure reported by the literature.[2] Briefly, a re-sealable flask was charged with PAF-1 (400.0 mg), paraformaldehyde (96%, 2.0 g), glacial AcOH (99.7%, 12.0 ml), H_3PO_4 (85%, 6.0 ml) and conc. HCl (36～38%, 45.0 ml). The flask was sealed and heated to 90 °C for 3 days. After the reaction, the solid was filtered, washed with water and methanol, and dried under vacuum overnight to afford PAF-1-CH_2Cl, as a yellow powder (yield: 96%). Elemental Analysis: C: 72.65%; H: 4.25%. ^{13}C CP-MAS NMR: 147, 138, 132, 66 and 53 ppm. IR: 1265, 1487 cm^{-1}.

Synthesis of PAF-1-TMA. PAF-TMA was prepared using the above same procedure by using trimethylamine (TMA, 98%) instead of NDMB (N,N-dimethyl-butylamine). The yield of PAF-1-TMA is 94%. Elemental Analysis: C: 50.42%; H: 7.16 %; N: 4.22 %. ^{13}C CP-MAS NMR: 146, 138, 129, 67, 53 and 45 ppm. IR: 1676, 2355, 2970 cm^{-1}.

Synthesis of PAF-SE. PAF-SE was prepared using the above same procedure by using sodium ethoxide (SE, 98%) instead of NDMB. The yield of PAF-1-NDMH is 95%. Elemental Analysis: C: 82.53%; H: 7.43%. ^{13}C CP-MAS NMR: 146, 139, 129, 71, 66 and 46 ppm. IR: 1093, 2864, 2972 cm^{-1}.

Synthesis of β-cyclodextrin (β-CD)-based polymer network (DFB-CDP). DFB-CDP was prepared according to previous work.[3] A 20 mL flame dried scintillation vial loaded with a magnetic stir bar was charged with β-Cyclodextrin (98%, 400 mg, 0.352 mmol), decafluorobiphenyl (98%, 353 mg, 1.056 mmol), K_2CO_3 (99%, 613 mg, 4.435 mmol) and dry *N*-methylpyrrolidone (98%, 10 mL). The vial was then bubbled with N_2 gas for 10 min. The vial was sealed and heated to 85 ℃ and stirred at 500 rpm for 40 hours. After the reaction, the pink gel was isolated and activated by water soxhlet extraction for 1 day and methanol

soxhlet extraction for 1 day. DFB-CDP (yield: 71%). Elemental analysis: C, 43.19 %; H, 2.86 %; F, 24.28 %; N＜0.02 %. IR: 3330, 2930, 1460, 1020 cm^{-1}.

Section 2. Test of chloride content in PAF-1-NDMB

The acid digestion was used to analysis halide ion.[4] Typically, 50 mg PAF-1-NDMB and concentrated HNO_3 (5 mL) was and into digestion tank. The sealed digestion tank was heated in 160 ℃ for 24 h. After cooling into room temperature, the residual liquid was diluted with deionized water to a final volume of 100 ml. The content of Cl^- was detected by ion chromatograph. Furthermore, the Cl^- content in filtrate after PFOA adsorption was also tested by ion chromatograph.

Section 3. Nonlinear pseudo second order kinetics model

$$q_t = \frac{k_2 q_e^2 t}{1+k_2 q_e t} \qquad \text{(Equation S-1)}$$

Where t is the reaction time (min), k_2 (g mg^{-1} min^{-1}) are the adsorption rate constants of nonlinear nonlinear pseudo-second-order, q_t and q_e (mg g^{-1}) are the adsorption capacity at a certain time and equilibrium time, respectively.[5-6]

Section 4. Regeneration Experiments

PFOA loaded PAF-1-NDMB was regenerated by soaking in a 100 ml combination solution of MeOH and saturated NaCl (v:v = 1:1) for 24 hours and recovered by filtration. The residual solid was collected and dried under vacuum (80 ℃ for 12h), which was used as the absorbent for another adsorption experiments.

Section 5. Supplementary Tables

Supplementary Table 1. Cl^- concentrations after acid digestion of PAF-1-NDMP, PAF-1-NDMB, and PAF-1-NDMH.

	Test 1	Test 2	Test 3	Mean value
Sample Name	Cl^- (mg L^{-1})	Cl^- (mg L^{-1})	Cl^- (mg L^{-1})	Cl^- (mg L^{-1})
PAF-1-NDMP	37.28	36.92	37.25	37.15
PAF-1-NDMB	47.62	47.72	47.73	47.69
PAF-1-NDMH	39.31	39.34	39.19	39.28

Supplementary Table 2. Cl^- concentrations in the filtrate after PFOA adsorption by PAF-1-NDMP, PAF-1-NDMB, and PAF-1-NDMH.

	Test 1	Test 2	Test 3	Mean value
Sample Name	Cl^- (mg L^{-1})	Cl^- (mg L^{-1})	Cl^- (mg L^{-1})	Cl^- (mg L^{-1})
PAF-1-NDMP	31.96	31.90	31.78	31.88
PAF-1-NDMB	40.11	40.36	40.19	40.22
PAF-1-NDMH	32.97	32.86	32.90	32.91

Supplementary Table 3. Cl^- concentrations after acid digestion of PFOA@PAF-1-NDMP, PFOA@PAF-1-NDMB, and PFOA@PAF-1-NDMH.

	Test 1	Test 2	Test 3	Mean value
Sample Name	Cl^- (mg L^{-1})	Cl^- (mg L^{-1})	Cl^- (mg L^{-1})	Cl^- (mg L^{-1})
PFOA@PAF-1-NDMP	5.11	4.96	5.02	5.03
PFOA@PAF-1-NDMB	7.10	7.18	7.08	7.12
PFOA@PAF-1-NDMH	6.26	6.35	6.32	6.31

Supplementary Table 4. The BET surface area, pore width, contact angle before and after adsorption of PFOA for PAF-1-NDMP, PAF-1-NDMB, and PAF-1-NDMH.

Samples	BET surface area (m^2 g^{-1})		Pore width (Å)		Contact angle (°)	
	Before adsorption	After adsorption	Before adsorption	After adsorption	Before adsorption	After adsorption
PAF-1-NDMP	602	14.6	5.6, 12.6	5.3	38.2	120.0
PAF-1-NDMB	108	9.0	12.1, 22.8	9.4	34.9	123.8
PAF-1-NDMH	50	7.1	12.6, 21.6	10.9	36.3	88.4

Supplementary Table 5. Parameters of linear and nonlinear pseudo second order kinetics model of the adsorbents.

Adsorbents	linear pseudo second order kinetics model		nonlinear pseudo second order kinetics model	
	k_2 (g mg^{-1} h^{-1})	R^2	k_2 (g mg^{-1} h^{-1})	R^2
PAF-1-NDMP	733.10	1.0000	5749.07	0.9989
PAF-1-NDMB	24000.00	1.0000	21166.94	0.9999
PAF-1-NDMH	1017.61	1.0000	14362.06	0.9999
PAF-1	17.15	0.9959	36.06	0.9701
AC	20.29	0.9939	32.90	0.9627
DFB-CDP	31.18	0.9993	63.04	0.9049

Supplementary Table 6. The shifts in IR spectra, and ^{13}C NMR spectra before and after adsorption of PFOA for PAF-1, PAF-1-NDMP, PAF-1-NDMB, and PAF-1-NDMH.

Samples	IR Spectra (cm^{-1})	
	Before adsorption	After adsorption
PAF-1	-	-
PAF-1-NDMP	1631.20	1636.57
PAF-1-NDMB	1676.00	1683.02
PAF-1-NDMH	1728.15	1733.93

Supplementary Table 7. The comparison of binding energy for PAF-1-NDMP, PAF-1-NDMB, and PAF-1-NDMH by density functional theory (DFT) calculation.

System	Binding Energy (eV)
PAF-1-NDMP	-0.285
PAF-1-NDMB	-0.290
PAF-1-NDMH	-0.303

Supplementary Table 8. The comparison of cost performance for PAF-1-NDMB with benchmark material of DFB-CDP.

Materials	Price \$ g^{-1}	PFOA uptake capacity mg g^{-1}	Cost for absorbing 1 g PFOA \$
DFB-CDP	6.45	62.50	103.28
PAF-1-NDMB	83.98	2000.00	41.99

The prices used for cost calculations are from Sigma-Aldrich or Fisher Scientific.

Section 6. Supplementary Figures

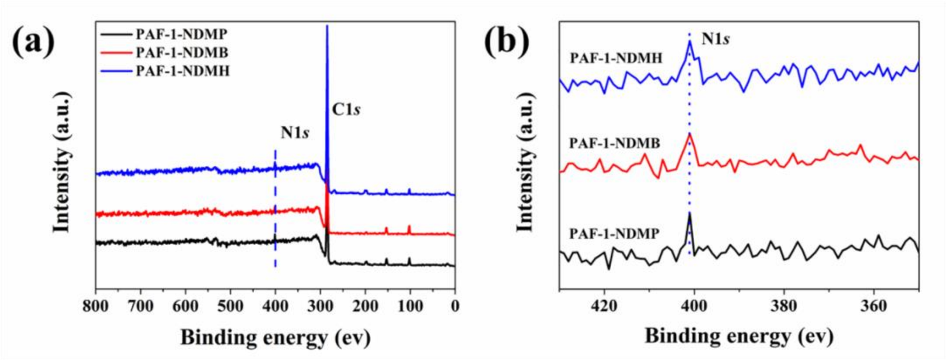

Supplementary Fig. 1. (a) XPS survey spectra of PAF-1-NDMP, PAF-1-NDMB, and PAF-1-NDMH; (b) XPS spectra of N1*s* of PAF-1-NDMP, PAF-1-NDMB, and PAF-1-NDMH.

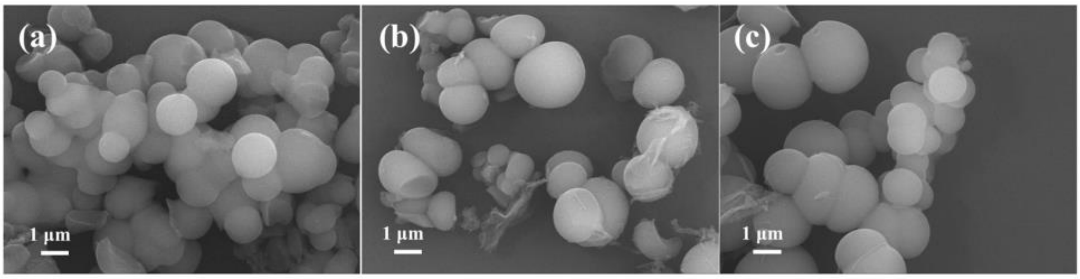

Supplementary Fig. 2. The SEM images of (a) PAF-1-NDMP, (b) PAF-1-NDMB, (c) PAF-1-NDMH.

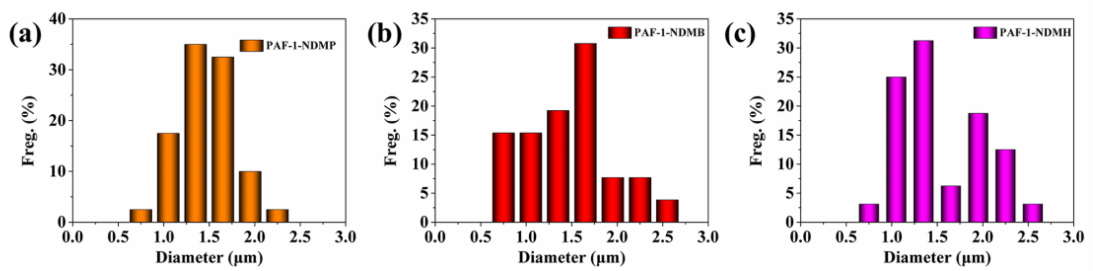

Supplementary Fig. 3. The particle size distribution calcalated in SEM images of (a) PAF-1-NDMP, (b) PAF-1-NDMB, (c) PAF-1-NDMH.

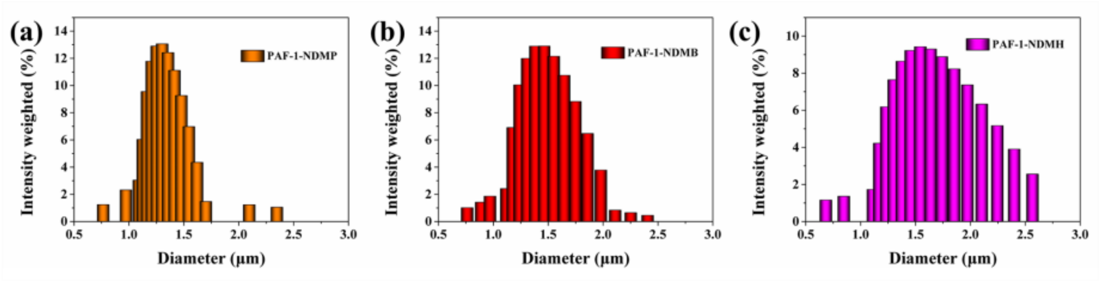

Supplementary Fig. 4. The particle size distribution (tested by dynamic laser light scattering DLS) of (a) PAF-1-NDMP, (b) PAF-1-NDMB, (c) PAF-1-NDMH.

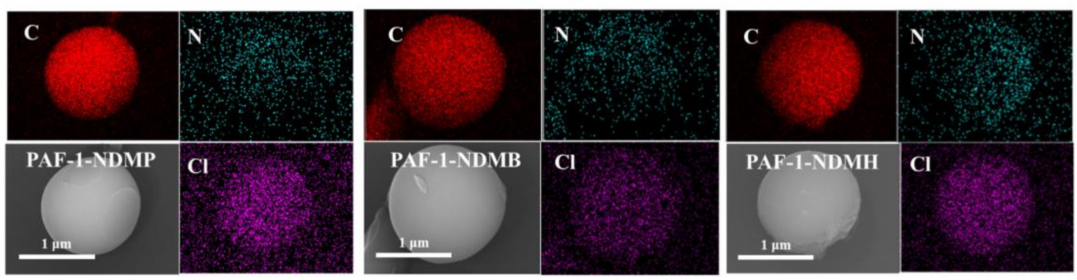

Supplementary Fig. 5. Energy-dispersive spectroscopy (EDS) mapping of PAF-1-NDMP, PAF-1-NDMB, and PAF-1-NDMH.

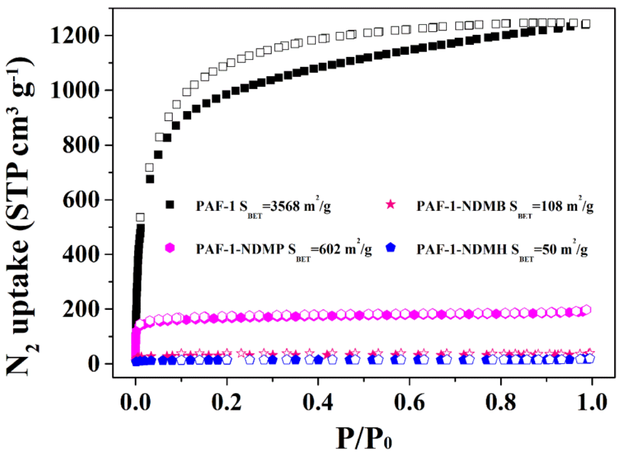

Supplementary Fig. 6. Nitrogen adsorption isotherms for PAF-1, PAF-1-NDMP, PAF-1-NDMB, and PAF-1-NDMH, and collected at 77 K. Filled symbols denote adsorption, and empty symbols denote desorption.

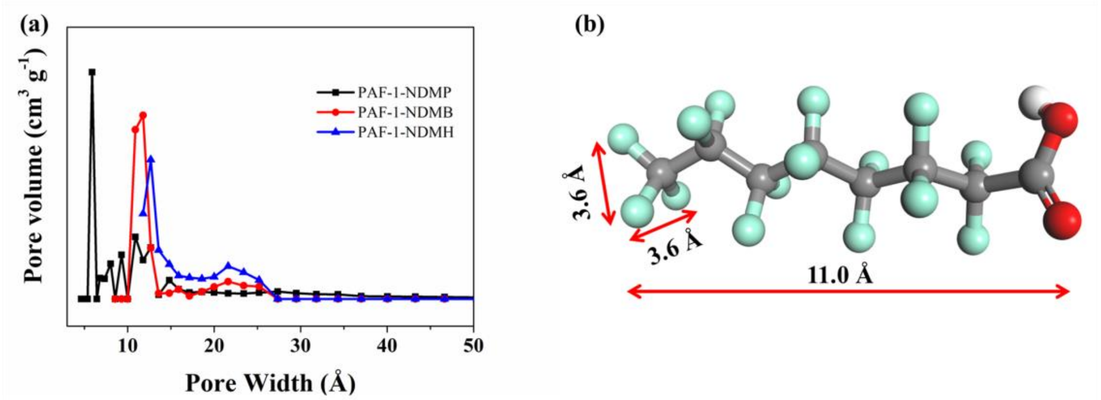

Supplementary Fig. 7. (a) Pore size distributions of PAF-1-NDMP, PAF-1-NDMB, and PAF-1-NDMH, (b) Dimension of PFOA molecule that drew and caculated (use forcite calculation) by material studio.

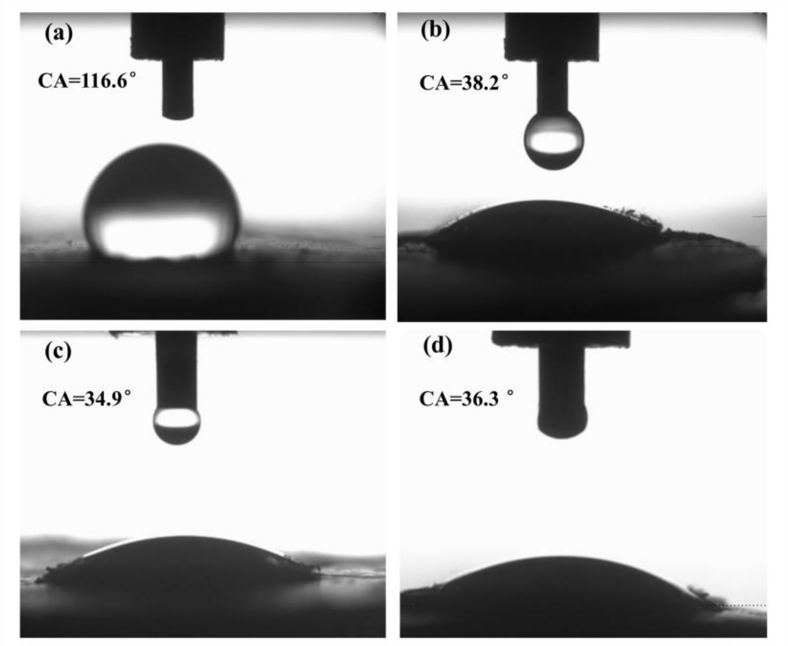

Supplementary Fig. 8. Water contact angle of (a) PAF-1, (b) PAF-1-NDMP, (c) PAF-1-NDMB, (d) PAF-1-NDMH.

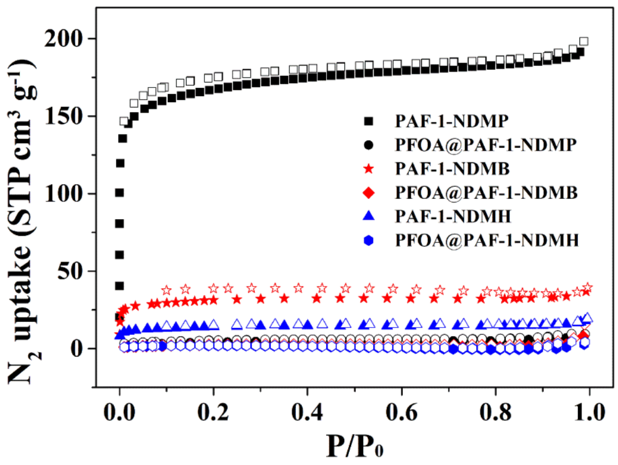

Supplementary Fig. 9. Nitrogen adsorption isotherms for PAF-1-NDMP, PFOA@PAF-1-NDMP, PAF-1-NDMB, PFOA@PAF-1-NDMB, PAF-1-NDMH and PFOA@PAF-1-NDMH collected at 77 K. Filled symbols denote adsorption, and empty symbols denote desorption.

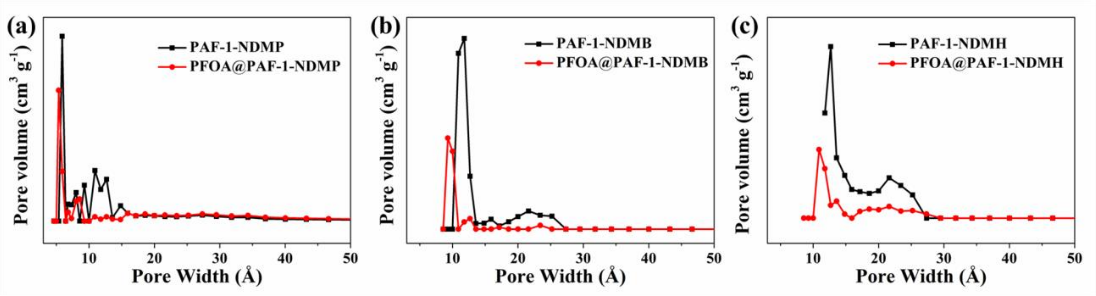

Supplementary Fig. 10. Pore size distributions of (a) PAF-1-NDMP and PFOA@PAF-1-NDMP, (b) PAF-1-NDMB and PFOA@PAF-1-NDMB, (c) PAF-1-NDMH and PFOA@PAF-1-NDMH.

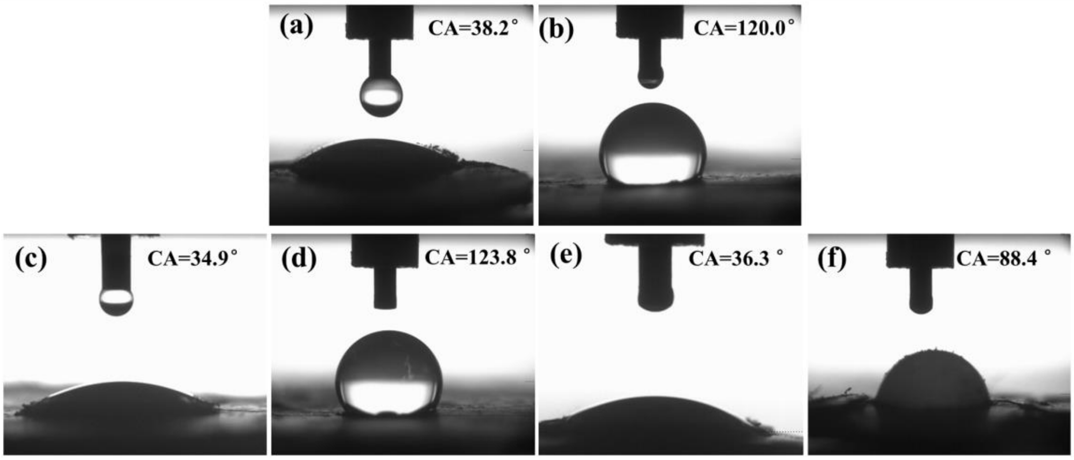

Supplementary Fig. 11. Water contact angle of (a) PAF-1-NDMP, (b) PFOA@PAF-1-NDMP, (c) PAF-1-NDMB, (d) PFOA@PAF-1-NDMB, (e) PAF-1-NDMH, and (f) PFOA@PAF-1-NDMH.

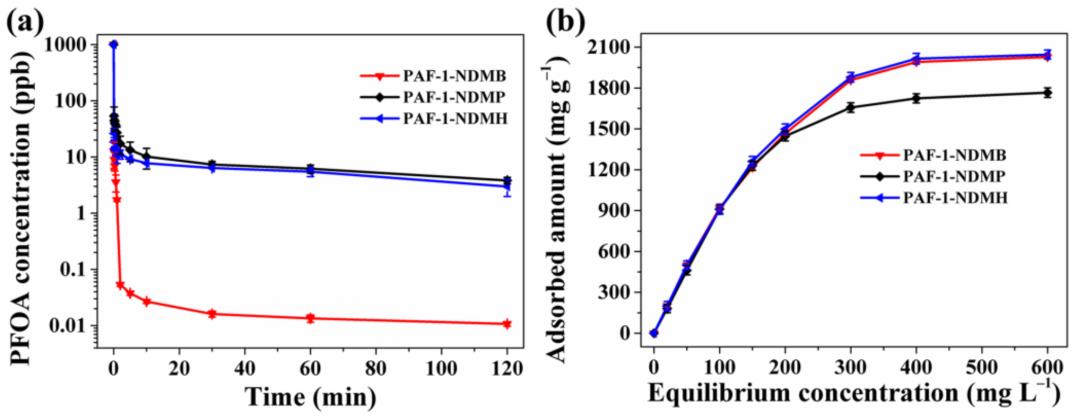

Supplementary Fig. 12. (a) PFOA sorption kinetics of PAF-1-NDMP, PAF-1-NDMB, and PAF-1-NDMH. (b) Plotting of equilibrium PFOA adsorption capacity as a function of equilibrium PFOA concentration. All the error bars in this figure represent the standard deviation (SD, n = 3 independent experiments), data are presented as mean values ± SD.

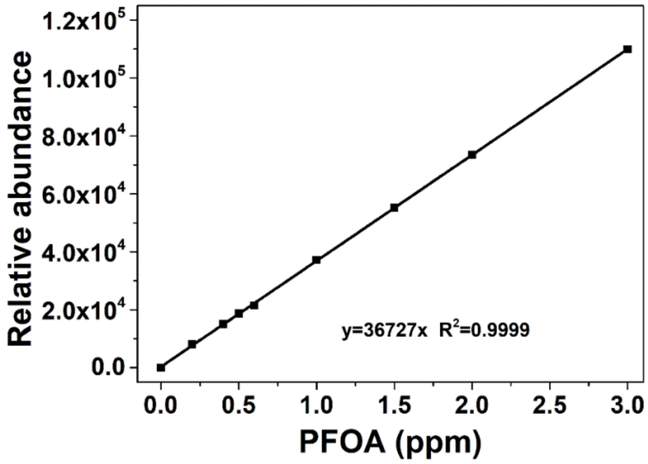

Supplementary Fig. 13. A representative standard curve showing the relative abundance of different concentrations of PFOA.

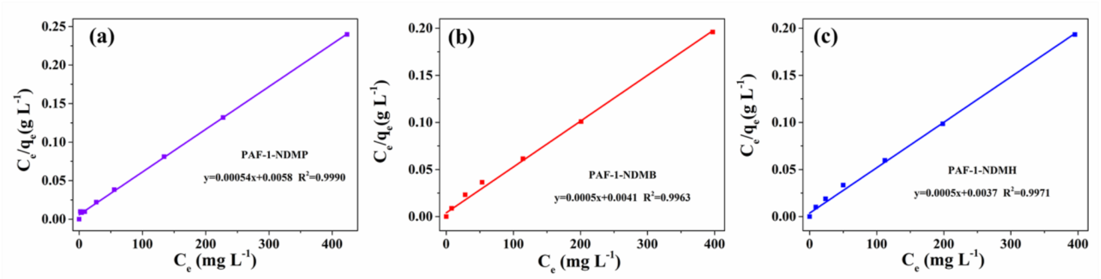

Supplementary Fig. 14. A representative standard curve showing the relative abundance of different concentrations of PFOA. Equilibrium PFOA uptake results of (a) PAF-1-NDMP, (b) PAF-1-NDMB, (c) PAF-1-NDMH from PFOA adsorption experiments, plotted according to the equation for the linearized Langmuir model.

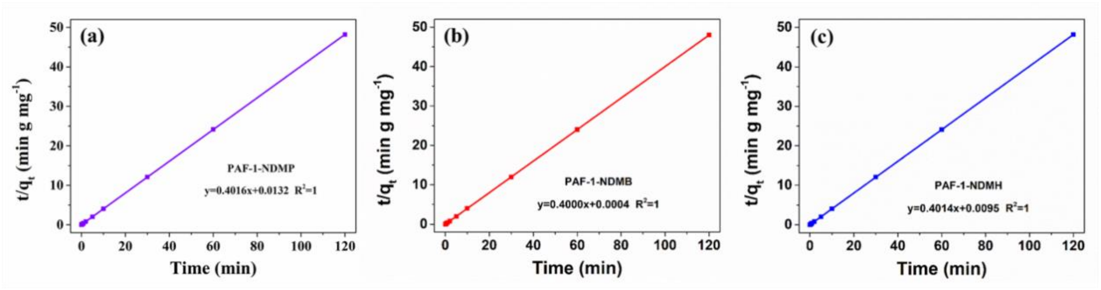

Supplementary Fig. 15. Linear pseudo-second-order kinetic plot of different samples for PFOA adsorption (PFOA concentration: 1000 ppb). (a) PAF-1-NDMP, (b) PAF-1-NDMB, (c) PAF-1-NDMH.

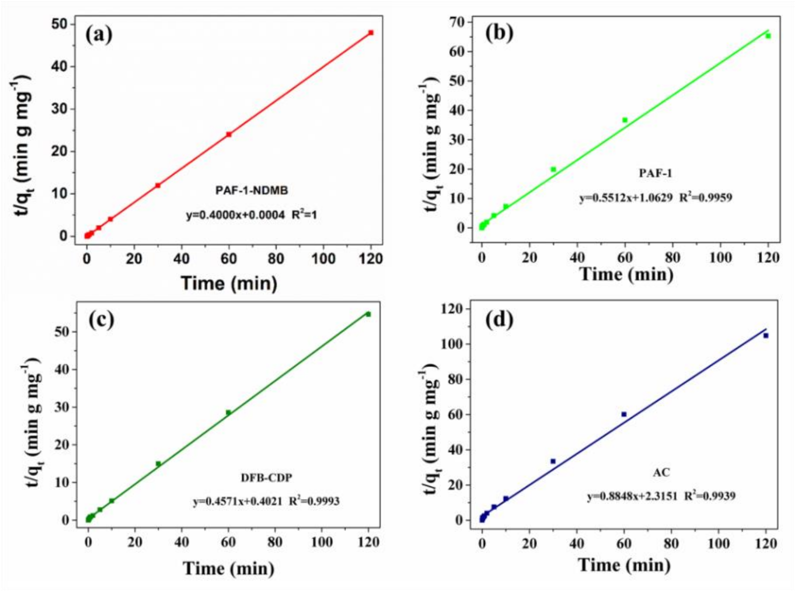

Supplementary Fig. 16. Linear pseudo-second-order kinetic plot of different samples for PFOA adsorption (PFOA concentration: 1000 ppb). (a) PAF-1-NDMB, (b) PAF-1, (c) DFB-CDP, (d) AC.

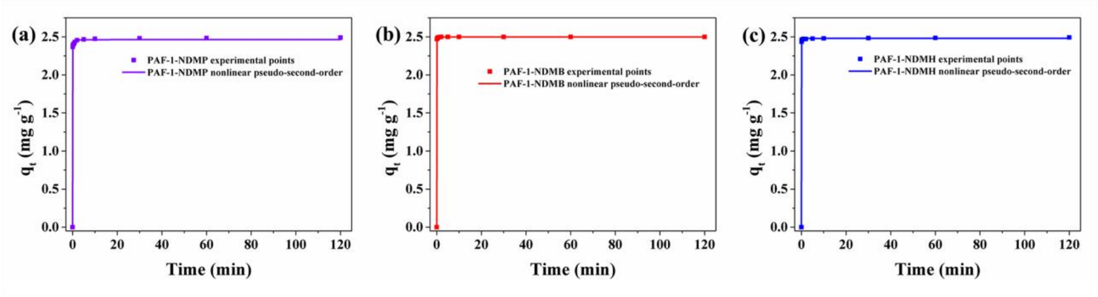

Supplementary Fig. 17. Nonlinear pseudo-second-order kinetic plot of different samples for PFOA adsorption (PFOA concentration: 1000 ppb). (a) PAF-1-NDMP, (b) PAF-1-NDMB, (c) PAF-1-NDMH.

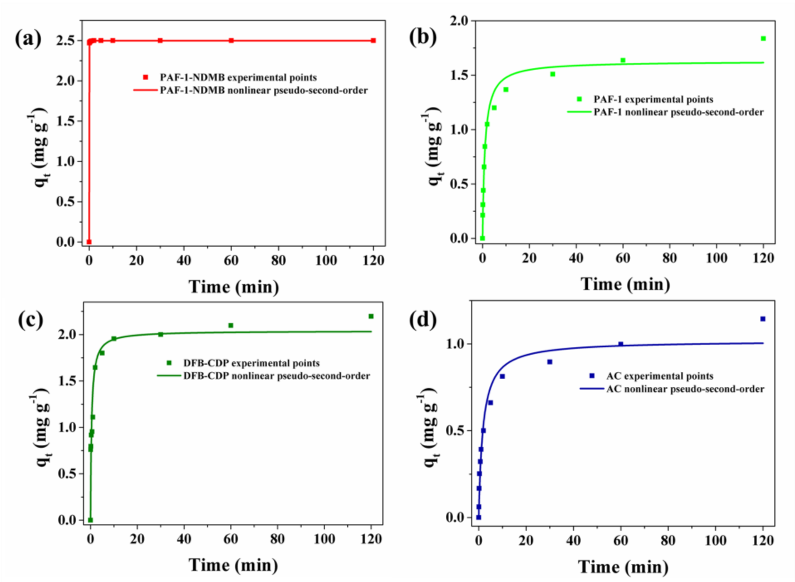

Supplementary Fig. 18. Nonlinear pseudo-second-order kinetic plot of different samples for PFOA adsorption (PFOA concentration: 1000 ppb). (a) PAF-1-NDMB, (b) PAF-1, (c) DFB-CDP, (d) AC.

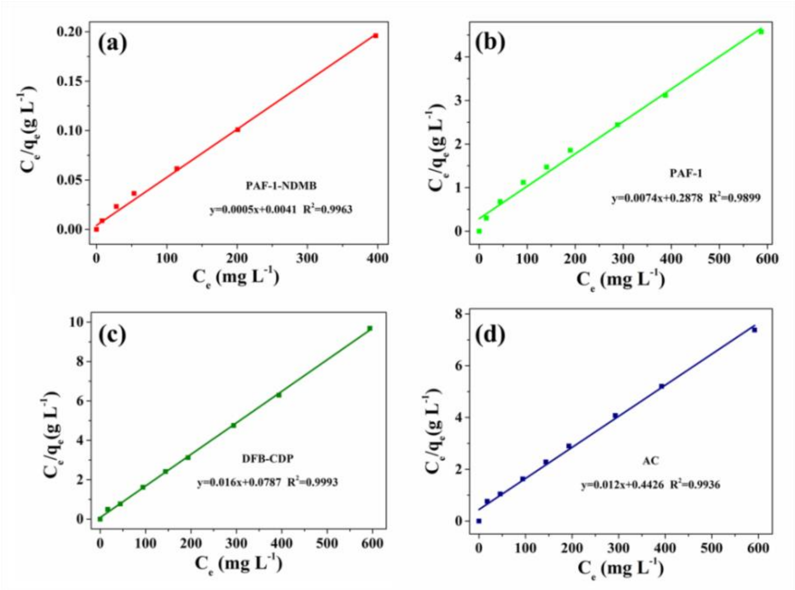

Supplementary Fig. 19. Equilibrium PFOA uptake results of (a) PAF-1-NDMB, (b) PAF-1, (c) DFB-CDP, (d) AC from PFOA adsorption experiments, plotted according to the equation for the linearized Langmuir model.

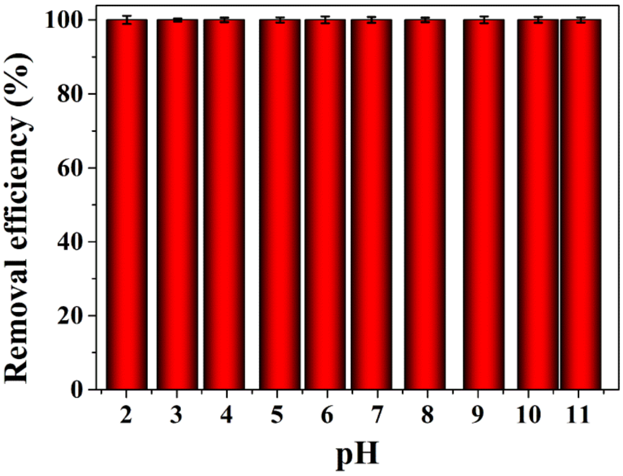

Supplementary Fig. 20. Adsorption performance of PFOA by the PAF-1-NDMB at different pH conditions. (Initial concentrations: [PFOA] = 1000 ppb, dosage of PAF-1-NDMB = 0.02 g; adsorption time: 2 h). All the error bars in this figure represent the standard deviation (n = 3 independent experiments), data are presented as mean values ± SD.

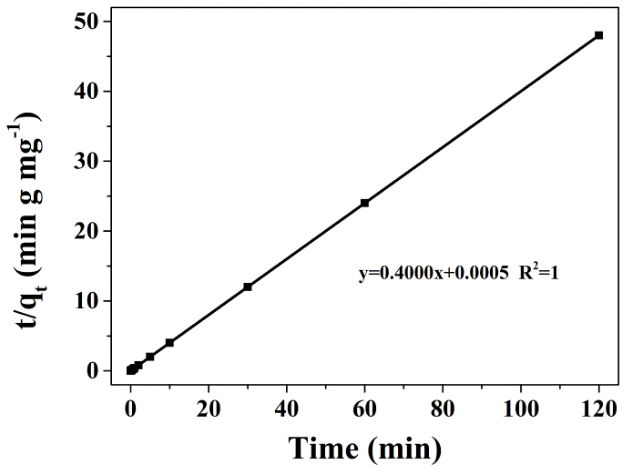

Supplementary Fig. 21. Linear pseudo-second-order kinetic plot of different samples for PAF-1-NDMB adsorption of PFOS (PFOS concentration: 1000 ppb).

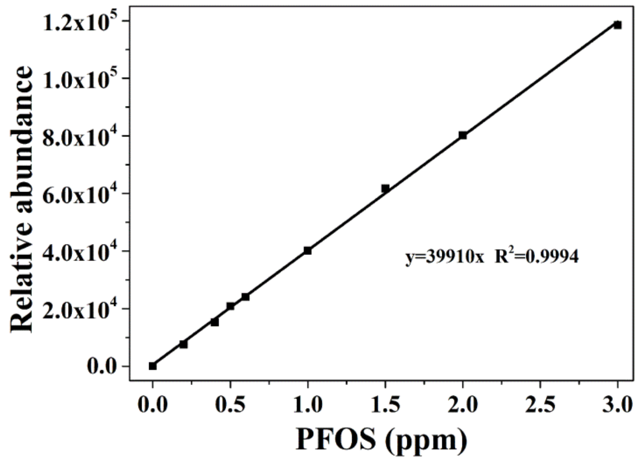

Supplementary Fig. 22. A representative standard curve showing the relative abundance of different concentrations of PFOS.

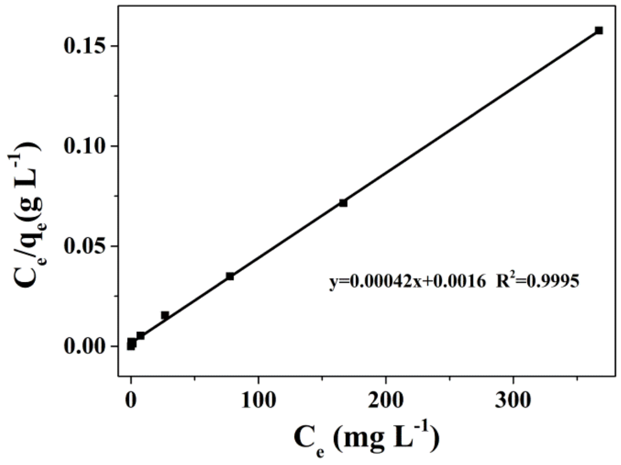

Supplementary Fig. 23. Equilibrium PFOS uptake results from PFOS adsorption experiments, plotted according to the equation for the linearized Langmuir model.

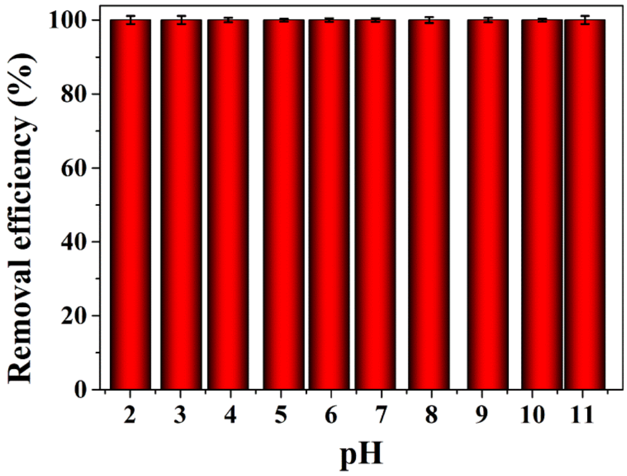

Supplementary Fig. 24. Adsorption performance of PFOS by the PAF-1-NDMB at different pH conditions. (Initial concentrations: [PFOS] = 1000 ppb, dosage of PAF-1-NDMB = 0.02 g; adsorption time: 2 h). All the error bars in this figure represent the standard deviation (n = 3 independent experiments), data are presented as mean values ± SD.

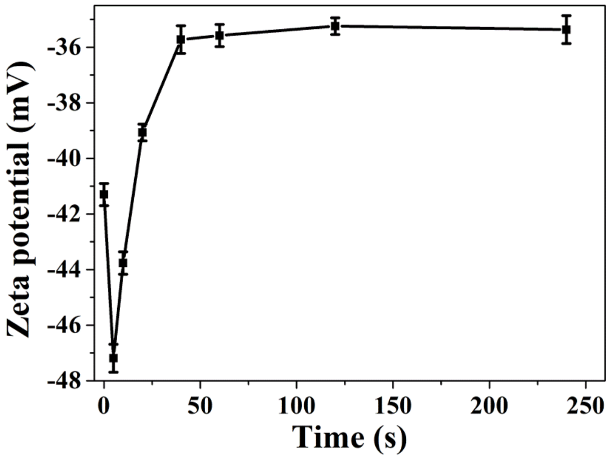

Supplementary Fig. 25. Zeta potential of PAF-1-NDMB after the exposure to a 1000 ppb PFOA solution. All the error bars in this figure represent the standard deviation (n = 3 independent experiments), data are presented as mean values ± SD.

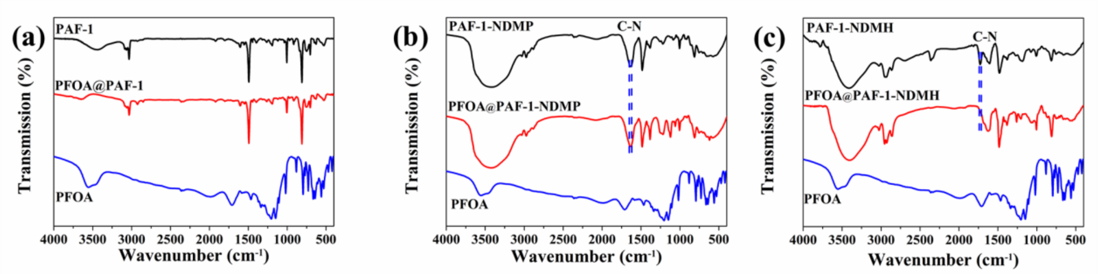

Supplementary Fig. 26. (a) FT-IR spectra of PFOA, PFOA@PAF-1, and PAF-1; (b) FT-IR spectra of PFOA, PFOA@PAF-1-NDMP, and PAF-1-NDMP; (c) FT-IR spectra of PFOA, PFOA@PAF-1-NDMH, and PAF-1-NDMH.

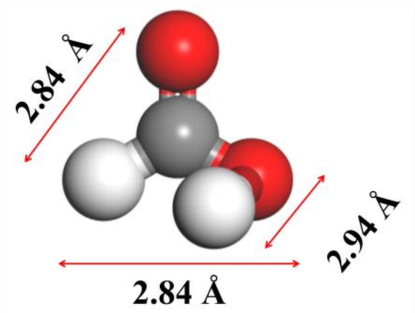

Supplementary Fig. 27. Dimension of FA molecule that drew and caculated (use forcite calculation) by material studio.

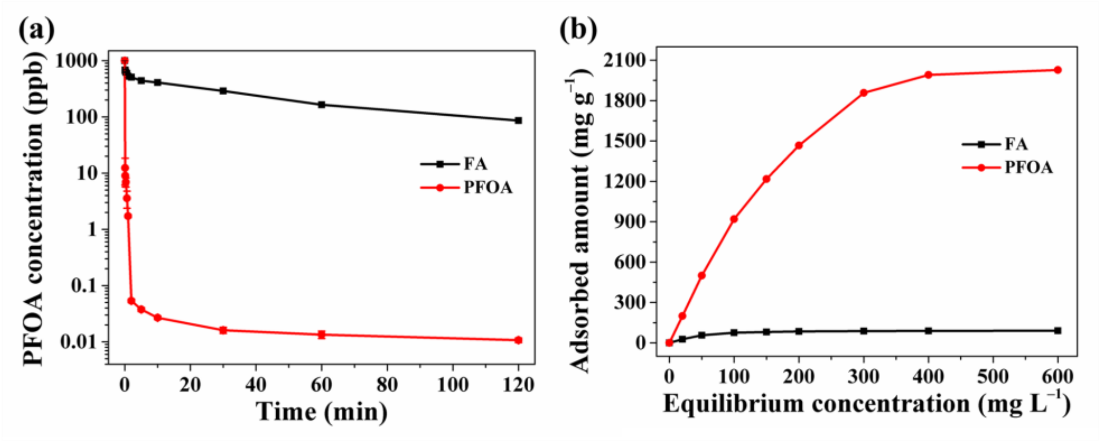

Supplementary Fig. 28. (a) The comparison of PFOA (red) and FA (Formic acid, black) sorption kinetics in PAF-1-NDMB. (b) The comparison of the equilibrium adsorption capacity for PFOA (red) and FA (black) as a function of equilibrium concentration. All the error bars in this figure represent the standard deviation (n = 3 independent experiments), data are presented as mean values ± SD.

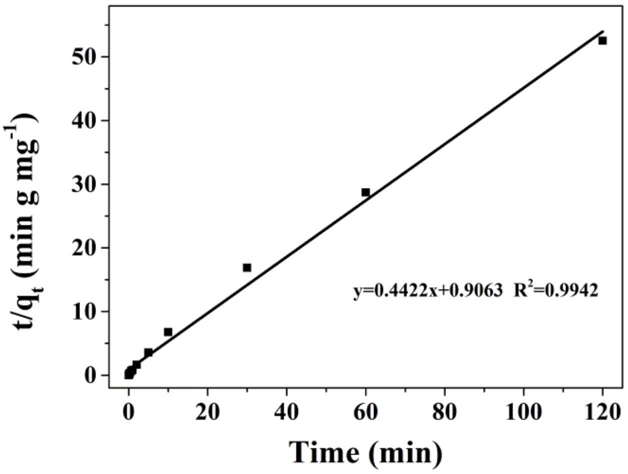

Supplementary Fig. 29. Linear pseudo-second-order kinetic plot for PAF-1-NDMB adsorption of FA (FA: 1000 ppb).

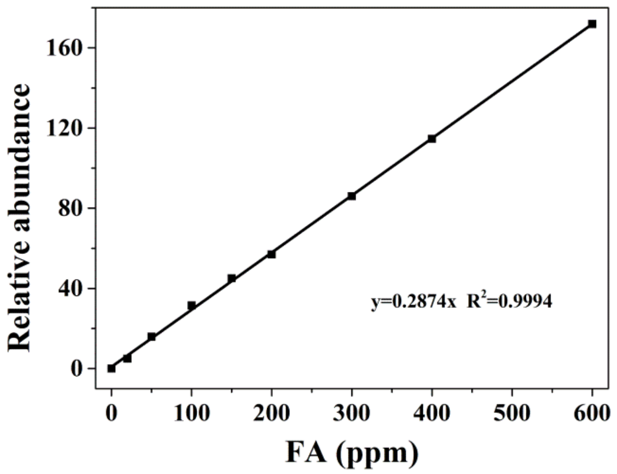

Supplementary Fig. 30. A representative standard curve showing the relative abundance of different concentrations of FA.

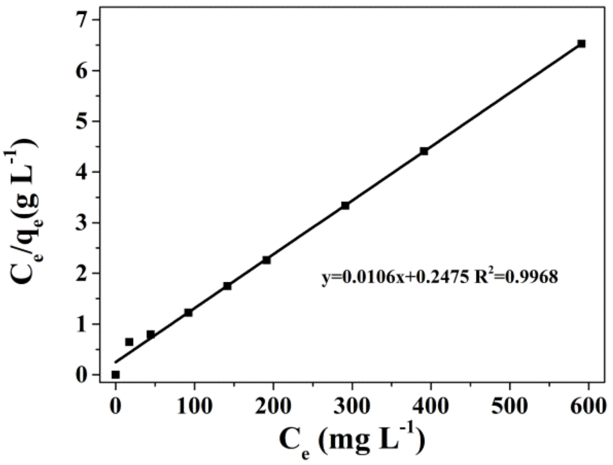

Supplementary Fig. 31. Equilibrium FA uptake results from experiments performed with FA solutions, plotted according to the equation for the linearized Langmuir model.

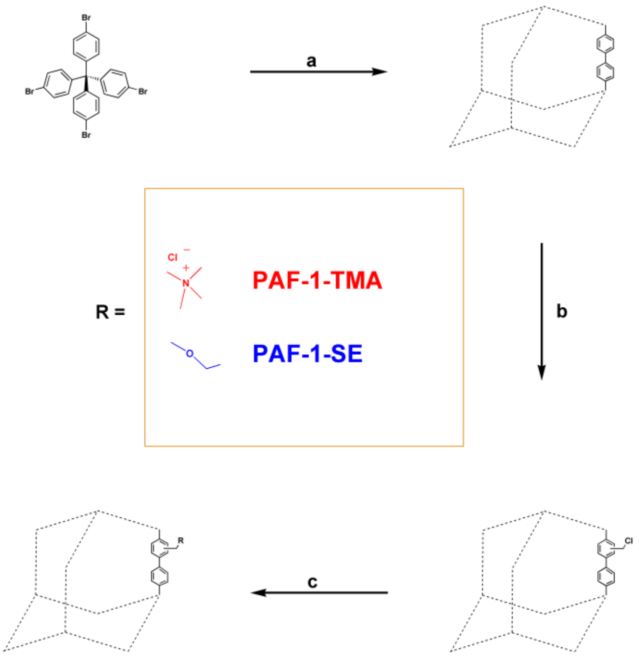

Supplementary Fig. 32. **General procedure for the synthesis of PAF-1-TMA, PAF-1-SE.** Reaction conditions: (a) Bis(1,5-cyclooctadiene)nickel(0), 1,5-cyclooctadiene, 2,2'-bipyridine, N,N-dimethylformamide, and tetrahydrofuran, room temperature; (b) Paraformaldehyde, AcOH, H_3PO_4, HCl, 90 ℃; and (c) trimethylamine (TMA) or sodium ethoxide (SE) in EtOH, 90 ℃.

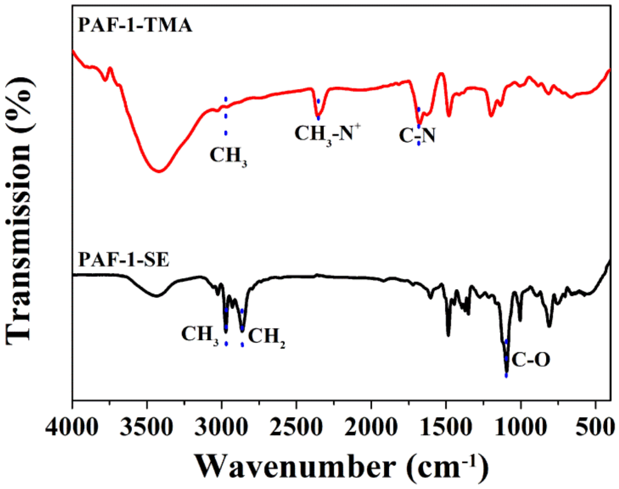

Supplementary Fig. 33. FT-IR spectra of PAF-1-SE, and PAF-1-TMA. FTIR spectroscopy showed that the appearance of new bands at 1093, and 1676cm^{-1} that were attributed to the C–O, and C-N of PAF-1-SE, and PAF-1-TMA, respectively, which indicated the successfully immobilization of functionalized groups onto PAF-1 framework.

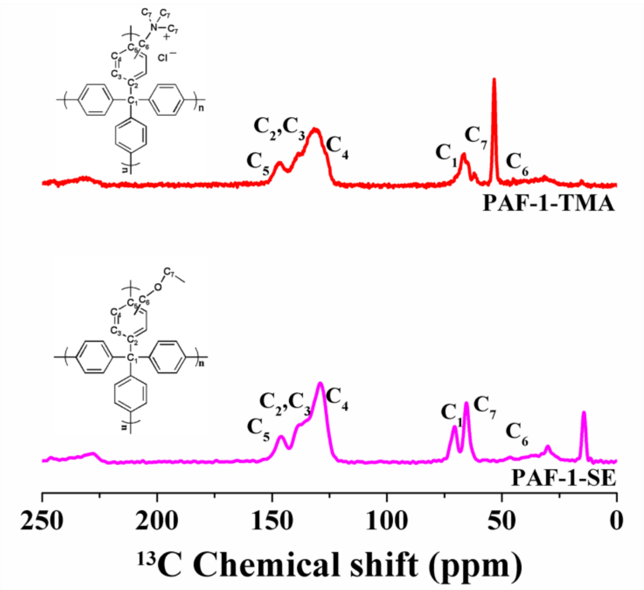

Supplementary Fig. 34. Solid state ^{13}C cross-polarization MAS NMR spectra of PAF-1-SE and PAF-1-TMA. The new broad resonance at 66 ppm, and 53 ppm of solid-state ^{13}C NMR for PAF-1-SE, and PAF-1-TMA indicate the successful incorporation of SE, and TMA onto the PAF-1 framework.

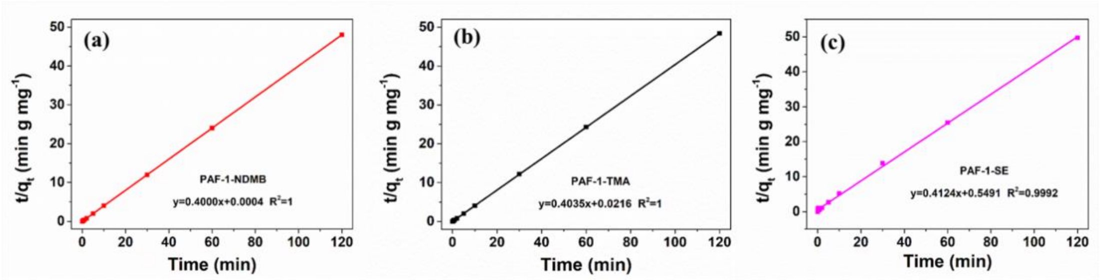

Supplementary Fig. 35. Linear pseudo-second-order kinetic plot of different samples for PFOA adsorption (PFOA concentration: 1000 ppb). (a) PAF-1-NDMB, (b) PAF-1-TMA, (c) PAF-1-SE.

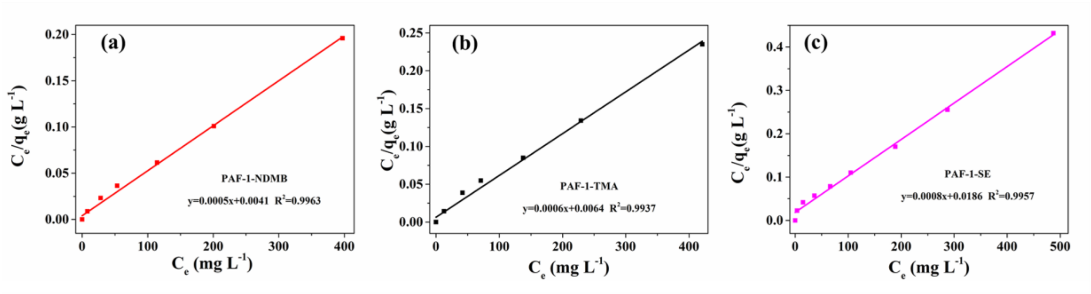

Supplementary Fig. 36. A representative standard curve showing the relative abundance of different concentrations of PFOA. Equilibrium PFOA uptake results of (a) PAF-1-NDMB, (b) PAF-1-TMA, (c) PAF-1-SE from PFOA adsorption experiments, plotted according to the equation for the linearized Langmuir model.

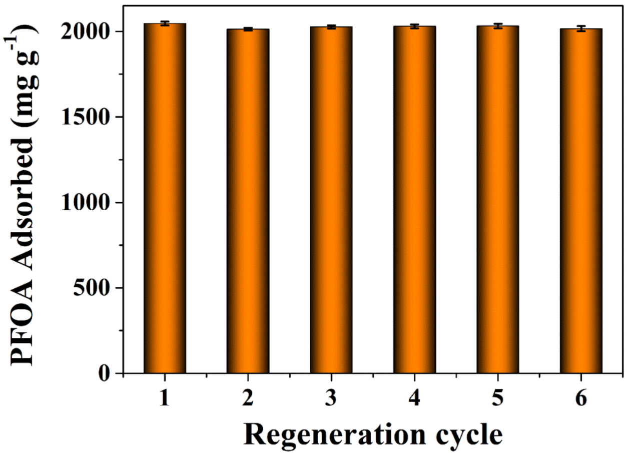

Supplementary Fig. 37. Regeneration cycles of PAF-1-NDMB by washing with the combined solution of MeOH and saturated NaCl. All the error bars in this figure represent the standard deviation (n = 3 independent experiments), data are presented as mean values ± SD.

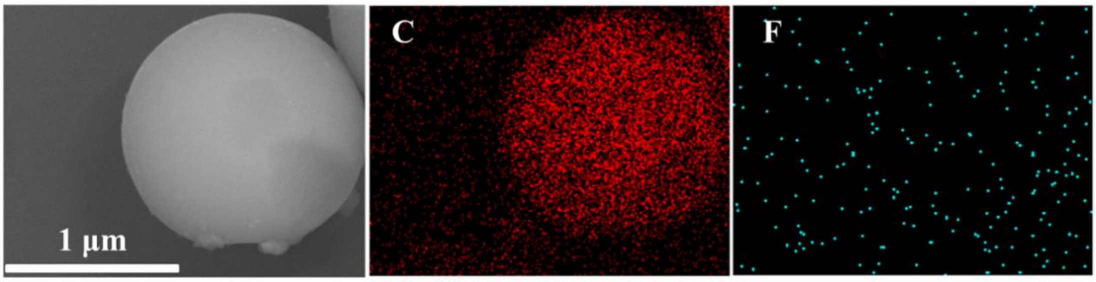

Supplementary Fig. 38. Energy-dispersive X-ray spectroscopy (EDS) mapping of the regenerated PFOA@PAF-1-NDMB. The EDS images showed that the F atoms are not detected after the regeneration, indicating that the complete removal of adsorbed PFOA molecules.

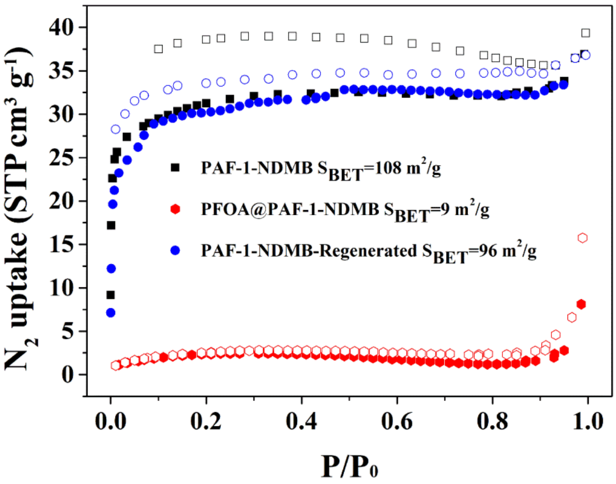

Supplementary Fig. 39. Nitrogen adsorption isotherms of PAF-1-NDMB, PFOA@PAF-1-NDMB, and PAF-1-NDMB-Regenerated, and collected at 77 K. Filled symbols denote adsorption, and empty symbols denote desorption.

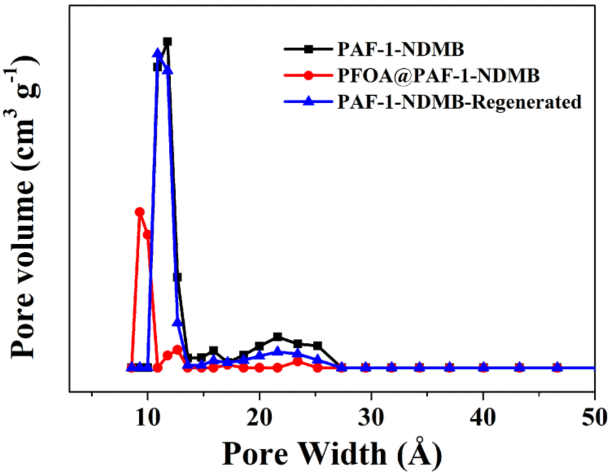

Supplementary Fig. 40. Pore size distributions of PAF-1-NDMB, PFOA@PAF-1-NDMB, and PAF-1-NDMB-Regenerated.

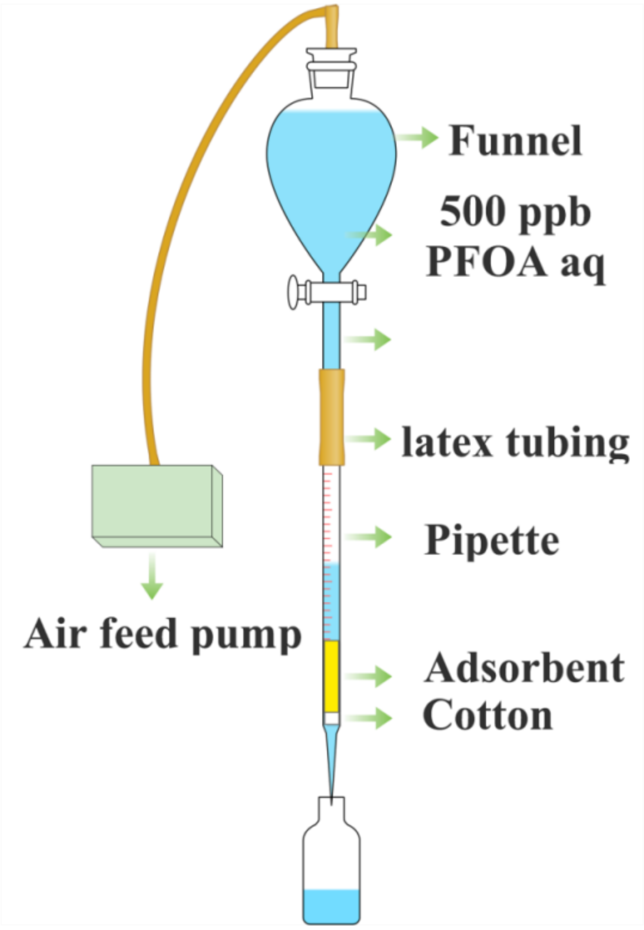

Supplementary Fig. 41. Scheme illustration of breakthrough experiment for PFOA adosprtion under HA (humic acid).

Section 7. References

1. Lu, W. et al. Porous polymer networks: synthesis, porosity, and applications in gas storage/separation. *Chem. Mater.* **22**, 964-5972 (2010).
2. Li, B., Zhang, Y., Ma, D., Shi, Z. & Ma, S. Mercury nano-trap for effective and efficient removal of mercury(II) from aqueous solution. *Nat. Commun.* **5**, 5537 (2014).
3. Xiao, L. et al. β-Cyclodextrin polymer network sequesters perfluorooctanoic acid at environmentally relevant concentrations. *J. Am. Chem. Soc.* **139**, 7689-7692 (2017).
4. Claret, F. et al. Natural iodine in a clay formation: Implications for iodine fate in geological disposals. *Geochim. Cosmochim. Acta* **74**, 16-29 (2010).
5. Simonin, J.-P. On the comparison of pseudo-first order and pseudo-second order rate laws in the modeling of adsorption kinetics. *Chem. Eng. J.* **300**, 254-263 (2016).
6. Kajjumba, G. W., Emik, S., Öngen, A., Kurtulus Özcan, H. & Aydın, S. Modelling of adsorption kinetic processes-errors, theory and application, *Adv. Sorption Process Appl.* 1-19 (2019).
